# Association between Sleep Quality and Depression Symptoms in Chinese College Students during the COVID-19 Lockdown Period

**DOI:** 10.3390/children9081237

**Published:** 2022-08-16

**Authors:** Cunjian Bi, Hongniu Lin, Jie Zhang, Zhimin Zhao

**Affiliations:** 1 School of Physical Education, Chizhou University, Chizhou 247000, China; 2 Sports Health Promotion Center, Chizhou University, Chizhou 247000, China; 3 Karamay No. 19 Primary School, Karamay 834009, China

**Keywords:** COVID-19, sleep quality, depression symptoms, college students

## Abstract

Background: The outbreak of COVID-19 has seriously threatened the health of people around the world. To prevent the spread of the epidemic, Chinese universities have implemented closed management of campuses. The implementation of restrictive measures has gradually caused changes in the quality of sleep and the psychological state of college students. In addition, college students are faced with the dual pressure of employment and study, and the psychological pressure is huge. Therefore, it is necessary to investigate sleep and depressive symptoms among college students. Methods: Using the method of stratified cluster sampling, 6695 college students were selected from three universities in Jiangxi, Anhui, and Xinjiang provinces from April to May 2022. The Chinese version of the Pittsburgh sleep quality index (PSQI) and the self-rating depression scale (SDS) were used for the survey. Hierarchical logistic regression analysis was used to analyze the relationship between the PSQI and the SDS. Results: Overall, during the outbreak of COVID-19, 69.0% of males and 73.5% of females had poor sleep quality among Chinese college students and the detection rate of depressive symptoms was 43.6% for males and 47.8% for females, respectively. Taking students with good sleep quality as references, after controlling for covariates, hierarchical logistic regression shows that Chinese college students with poor sleep quality have a higher OR value (OR = 12.0, 95%CI: 10.2~14.1, *p* < 0.001), especially in males (OR = 43.8, 95%CI:30.2~63.6, *p* < 0.001). For both males and females, the OR value of college students with the following characteristics was higher: rural college students (males, OR = 50.32, 95%CI: 32.50–77.93; females, OR = 8.03, 95%CI: 6.45–9.99), overweight college students (males, OR = 62.17, 95%CI: 19.47–198.53; females, OR = 16.67, 95%CI: 6.48–42.88), and college students drinking sugar-sweetened beverages (males, OR = 59.00, 95%CI: 36.96–94.18; females, OR = 8.16, 95%CI: 6.63–10.05) (*p* < 0.001). Conclusions: Poor sleep quality is associated with depressive symptoms among Chinese college students, especially college males. Our research suggests that it is necessary to consider the improvement of sleep quality and depressive symptoms among college students during the COVID-19 epidemic.

## 1. Introduction

College students aged 18–24 live through a period of psychological changes and sudden autonomy on the transition from high school to professional life, facing the dual pressure of employment and academics. Their psychological status during this period can impact their academic life and going forward as adults. The American Institute of Stress (AIS) statistics show that 80% of college students often suffer from a lack of sleep due to mental stress [1].

With the outbreak of COVID-19 in December 2019, quarantine was used as an important way to reduce human-to-human contact and prevent its spread [2]. However, quarantine may cause people to feel fearful, lonely, and even anxious, affecting their mental health [3,4,5]. It was reported that 32% of young people in the UK believed that COVID-19 made their mental health worse [6]. Studies of college students in France, Ethiopia, and Malaysia have shown that COVID-19 has a significant negative impact on the mental health of college students, resulting in a higher prevalence of depression and stress [7,8]. Studies have pointed out that young people and those who spend too much time thinking about the epidemic are at higher risk of mental illness [9].

Depression, which affected 264 million people worldwide before the COVID-19 outbreak, has seen a 25% increase in global depression prevalence in 2020, with young adults being the most affected [10]. Research shows that COVID-19 has continued to increase the detection rate of depression in various countries around the world, reaching a significantly higher level than before the outbreak [10,11]. An online survey of mental health of 4872 people over 18 years old covering 31 provinces and autonomous regions in China showed that 48.3% suffered from depression [12]. With the closure of schools and the cancellation of recreational and cultural activities, many teenagers missed out on opportunities to pursue happiness during the COVID-19 epidemic, inevitably causing depression [13]. A survey of 2031 US college students showed that 48.14% exhibited moderate to severe depression [14]. The evaluation of the mental health of Chinese college students showed that the level of depression has also increased [15,16]. The incidence of depression among 3881 college students in Guangdong was 21.16% [17], which was higher than that before the COVID-19 outbreak (19.33%) [18].

As an important physiological phenomenon of human beings, sleep has been described as the single most important health behavior. Sleep quality is a measurement that is related to sleep hygiene and poor sleep quality has been understood to have serious health consequences [19]. Further, studies have documented poor sleep quality among university students [20,21,22]. In China, a study also revealed a high prevalence of sleep problems among college students during the COVID-19 epidemic [23].

A systematic review suggested that the current literature supports a bidirectional relationship between sleep and depression [24]. An international study including more than 20,000 college students from 26 countries showed that there is a link between depression and sleep disorders among college students [25]. Research on the relationship between sleep and depression during the COVID-19 epidemic in China has mainly focused on children and adolescents and social adult groups, while limited studies have been conducted on Chinese college students. The present study aimed to estimate the association between sleep quality and depression symptoms in Chinese college students during the COVID-19 lockdown period.

## 2. Methods

### 2.1. Data Collection and Participants

**Figure 1:**
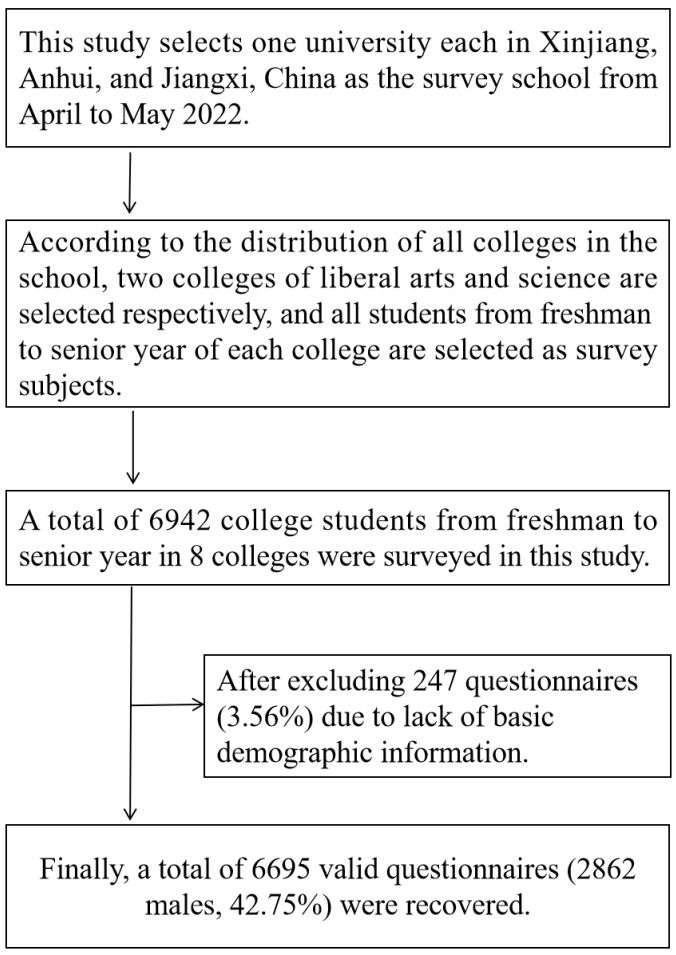
The specific sampling process of participants.

Three universities were selected from three provinces (Xinjiang, Anhui, and Jiangxi) in western, eastern, and central China. We adopted cluster sampling by the school after communicating with the school administrators. According to the distribution of the colleges in the selected university, two colleges were randomly selected, and all the students in the selected colleges meeting the inclusion criteria were selected as participants. The exclusion criteria for participation in this study were no mental illness and no family history of mental disorders. Participants’ inclusion criteria were only officially registered college students, who own a mobile phone, can communicate normally with people, and voluntarily chose to participate in this study. A total of 6942 college students from freshman to senior year in 8 colleges were surveyed in this study. After excluding 247 questionnaires (3.56%) due to a lack of basic demographic information, a total of 6695 valid data (2862 males, 42.75%) were obtained (Figure 1).

The written informed consent of the students was obtained before the investigation of this study, and the investigation was conducted using anonymous coding. This research investigation was approved by the Human Ethics Committee of Chizhou University on 15 January 2022 (202201045).

### 2.2. Procedure

An electronic questionnaire including basic information (age, gender, school, grade, class, urban or rural area, siblings, and parents’ educational level), depressive symptoms, and sleep quality was used for this study. Teachers distributed a quick response code to the students for scanning and the students completed the electronic questionnaires by using their cell phones in the presence of professional investigators.

### 2.3. Depressive Symptoms

The self-rating depression scale (SDS) was developed specifically to depression by Zung in 1965 [26,27] and its items were chosen based on factor analytical studies of depression symptoms. There is already evidence suggesting that the SDS score is a strong predictor of Patient Health Questionnaire depression diagnoses and can be used as a screener for depression [28]. This scale consists of 20 items, each of which is scored on a 1–4 scale, as “no or little time”, “sometimes”, “a lot of the time”, and “most of the time”. Items 2, 5, 6, 11, 12, 14, 16, 17, 18, and 20 are scored in reverse. The scores of each item are combined to obtain an approximate score of 20–80, and the approximate score is multiplied by 1.25 to obtain a standard score of 25–100. In this study, a standard score of <50 was considered as having no depressive symptoms; a standard score of ≥50 was considered as having depressive symptoms; 50–59 was considered as having mild depression; 60–69 was considered as having moderate depression; and ≥70 was considered as having severe depression.

### 2.4. Sleep Quality

As the classic tool for measuring sleep quality, the Pittsburgh sleep quality index has been developed into many versions around the world [29]. We used the Chinese version of the Pittsburgh sleep quality index (PSQI) [30] to measure sleep quality in the present study, which has been proven to be a tool with good reliability and validity to measure sleep quality for Chinese populations [31,32,33,34,35]. The scale consists of 7 dimensions, namely subjective sleep quality, sleep latency, sleep duration, habitual sleep efficiency, sleep disturbance, use of sleep medication, and daytime dysfunction. Each dimension is scored on a 0–3 scale, and the total score is 0–21 points. A score of ≤5 points was considered as good sleep quality, 6–7 as moderate, and ≥8 as sleep disturbance. In this study, a PSQI score of ≤5 was considered good sleep quality, and a score of >5 was considered poor sleep quality [36,37].

### 2.5. Covariates

Results from many studies have demonstrated that sleep quality or depression can be affected by body mass index (BMI) [38], physical activity [39], screen time [40], sugar-sweetened beverages [41], parents’ education level [42], and socioeconomic status (SES) [43,44]. Therefore, the present study takes these factors as covariates.

BMI was calculated by weight (kg)/height (m)^2^. The height and weight are carried out according to the test methods and instruments required by the National Student Physical Health Standard (NSPHS). The height is accurate to 0.1 cm and the weight is accurate to 0.1 kg [45]. The BMI results were divided, according to the standards in the Chinese adult overweight and obesity prevention and control guidelines [46], into underweight, normal, overweight, and obese. A BMI < 18.5 kg/m^2^ is defined as underweight; a 18.5 kg/m^2^ ≤ BMI < 24.0 kg/m^2^ is defined as normal; a 24.0 kg/m^2^ ≤ BMI < 28.0 kg/m^2^ is defined as overweight; a BMI ≥ 28.0 kg/m^2^ is defined as obese.

Physical Activity: The survey on physical activity was conducted using the short questionnaire of the International Physical Activity Scale (IPAQ), which has a total of 7 items. The subjects were asked about the time and frequency of physical activity of different intensities in the past 7 days to calculate the daily physical activity time. In this study, according to the recommended amount of international physical activity [34], moderate-to-high-intensity physical activity time (MVPA) ≥ 60 min per day was defined as a person who achieved the physical activity standard, “Yes”; on the contrary, those who do not meet the standard answered as “No”.

Screen Time: Screen time mainly investigates the screen time of subjects watching TV and playing on mobile phones and tablet computers in the past 7 days. It is divided into Monday to Friday on weekdays and Saturday and Sunday on weekends. The average daily screen time was calculated for the past 7 days. According to the relevant standard [47], this study divides screen time into two groups, ≤2 h/d and >2 h/d for statistical analysis.

Sugar-sweetened beverages (SSBs): SSBs are mainly used to investigate the consumption of sugar-sweetened beverages, such as cola, tea, and fruit juice in the past 7 days, including the frequency and amount of drinking. The calculation of the amount is based on a 330 mL bottle of ordinary cola. In this study, those who consumed at least 1 bottle of sugar-sweetened beverages in the past 7 days answered “Yes”, and those who did not answer “No”.

Parents’ education level: This study investigates father’s education and mother’s education, respectively, divided into three levels, namely elementary school and below, middle school, college, and above.

Socioeconomic status (SES): Indicators such as parents’ educational level, parents’ occupation, and family income are usually used in academia to measure the socioeconomic status of adolescents’ families. This study is classified according to the method of calculating the SES of the Program for International Student Assessment (PISA). Parents’ education level is scored according to the number of years of education, and the occupational classification is carried out according to the scoring standard in the International Socioeconomic Status Occupational Classification Index (ISEI) by Ganzeboom [48]. Because most teenagers do not know much about family income, family resources are used for indirect approximate measurement, including 9 household daily necessities such as computers and televisions. According to the standardized calculated SES score, it was divided into low (<15 th), medium (15–85 th), and high (>85 th) based on the percentile.

### 2.6. Statistical Analysis

Our analysis was stratified by gender since there is a difference in the detection rate of depressive symptoms between males and females. The detection rate of depressive symptoms in males and females was expressed as a percentage, and the different variables were compared with a chi-square test. The relationship between depressive symptoms and sleep quality for males and females was analyzed by subgroup analyses. Hierarchical logistic regression was conducted with three models: the Crude Model was conducted without adjustment; Model 1 was conducted after adjusting for age, urban or rural area, siblings, BMI, physical activity, screen time, and sugar-sweetened beverage consumption; based on Model 1, Model 2 included parents’ education level and SES as additional control variables. Analyses were presented as the odds ratio (OR) and 95% confidence intervals (95%CI). Additionally, the strength of the correlation between independent variables and depressive symptoms was evaluated. *P* < 0.05 was considered to be statistically significant. Statistical analysis was performed using SPSS 25.0 software (IBM, Armonk, NY, USA).

## 3. Results

Table 1 shows the detection rates of depressive symptoms in different variable populations stratified by gender. A total of 6695 subjects were investigated in this study, including 2862 males and 3833 females. In general, males with poor sleep quality accounted for 69.0% and females accounted for 73.5%; the detection rate of depressive symptoms was 43.6% for males and 47.8% for females. Our study also showed that compared with college students with poor sleep quality, the detection rate of depressive symptoms was higher than that of college students with better sleep quality.

Table 2 shows the results of logistic regression analysis for males and females after controlling for covariates. Compared with the good sleep quality group of college students, males with poor sleep quality (OR = 44.33, 95%CI: 30.63–64.16) and females (OR = 7.30, 95%CI: 6.09–8.76) had a higher risk of developing depressive symptoms (*p* < 0.001). Compared with other independent variables, sleep quality had the highest OR value for depressive symptoms.

Table 3 shows the result of the hierarchical logistic regression analysis after controlling for relevant covariates. Except for sleep latency and sleep disturbance in females, there was a dose-response relationship between subjective sleep quality, sleep duration, habitual sleep efficiency, use of sleep medication, daytime dysfunction, and depressive symptoms in males and females (*p* < 0.05). Among all factors, the OR value of subjective sleep quality in college males and females with depressive symptoms is the largest, and the subjective sleep quality is very bad for college males (OR = 3516.28, 95%CI: 1278.92–9667.67) and females (OR = 2557.61, 95%CI: 913.87–7157.92) had a much higher risk of developing depressive symptoms compared with students with very good subjective sleep quality (*p* < 0.001).

Table 4 is a subgroup analysis of the relationship between sleep quality and depressive symptoms in college students with different variables. In general, the OR value of males was higher than that of females. In terms of age group, among college students with poor sleep quality, 21-year-old college males (OR = 78.33, 95%CI: 28.60–214.56) and college females (OR = 8.29, 95%CI: 5.34–12.88) had the highest OR values (*p* < 0.001). In terms of other variables, for both males or females, the OR values of college students with the following characteristics were higher: rural college students (males, OR = 50.32, 95%CI: 32.50–77.93; females, OR = 8.03, 95%CI: 6.45–9.99), overweight college students (males, OR = 62.17, 95%CI: 19.47–198.53; females, OR = 16.67, 95%CI: 6.48–42.88), and college students drinking sugar-sweetened beverages (males, OR = 59.00, 95%CI: 36.96–94.18; females, OR = 8.16, 95%CI: 6.63–10.05) (*p* < 0.001). 

Taking students with good sleep quality as references, after controlling for covariates, hierarchical logistic regression shows that Chinese college students with poor sleep quality have a higher OR value (OR = 12.0, 95%CI: 10.2~14.1, *p* < 0.001), especially in males (OR = 43.8, 95%CI:30.2~63.6, *p* < 0.001) (Table 5).

## 4. Discussion

Our study found that during the COVID-19 epidemic, Chinese college students had a higher prevalence of depression and poor sleep quality. The results of this study showed that males with poor sleep quality accounted for 69.0% and females accounted for 73.5%, while the rate of poor sleep quality among college students during the study of new coronavirus at home and abroad was between 5% and 70% [14,49,50]. It can be seen that Chinese college males in the present study already score close to the upper limit, and the proportion of female students exceeds this ratio, indicating that the overall sleep quality of Chinese college students is poor during the COVID-19 epidemic. In terms of gender and sleep quality, as in previous studies, female college students have longer sleep latency, more frequent awakenings, and poorer sleep quality than male college students [51]. This study shows an association between epidemic exposure and sleep quality. Chinese campus epidemic prevention measures will continue to affect the physical and mental health of college students, increasing sleep problems. The reduction in offline gathering activities during the epidemic has led to a reduction in the frequency of college students participating in social activities, sports, and art activities. Decreased physical activity and social relationships may lead to increased time spent on electronic devices, increased online socialization, and increased likelihood of sleepiness and naps, which may individually or collectively affect bedtime and sleep quality [52].

Previous studies have shown that approximately 10–25% of college students suffer from depression, and many of them experienced their first depression during college [53]. According to the Chinese norm, the cut-off value of the SDS score is 33.46 ± 8.55 points [54]. Our study shows that the detection of depressive symptoms is 43.6% for males and 47.8% for females. The detection rate of depression for Chinese college students in our survey is higher than that before the pandemic (19%) [18] and for college students from 15 countries (31.2%) during the epidemic [55]. However, this result is lower than the depression prevalence rate of 45% among 1134 college students in Pakistan and is far lower than the 80.57% of 2031 undergraduate and graduate students in the United States (approximately 48% were in the moderate to severe range) [14]. The reason for this difference may lie in the different populations and different test scales used. The proportion of male and female students suffering from depression exceeds the previous survey report by the American University Health Association (45% for females and 36% for males) [56]. Our findings, therefore, demonstrate that the COVID-19 outbreak has a significant impact on the mental health of Chinese college students.

Our findings are consistent with previous studies showing that poor sleep quality is associated with depression [57,58]. In addition, Nyer’s study showed that college students with depressive symptoms and sleep disturbances experienced a greater burden of comorbidities and a greater loss of physical and cognitive function than those without sleep disturbances [59]. Although empirical assessment of sleep quality can be complex, the PSQI is often used in research as the sole measure of sleep quality [60]. Additionally, there is ample evidence that sleep disturbance is a precursor to the development of depression [59]. A study on the COVID-19 outbreak in Italy investigated sleep problems among online students during home isolation, and the results showed that students with more severe depressive symptoms during the epidemic had greater changes in sleep quality [61]. A study on the impact of the epidemic on the sleep quality of college students in China showed that the sleep quality of college students during the epidemic was worse than that before the epidemic, and depressive symptoms were related to sleep quality [62]. Numerous data suggest that dopaminergic and hypothalamic–pituitary–thyroid physiological effects may directly affect sleep quality [55]. In addition, poor sleep quality and depressive disorders are biologically associated with functional connectivity in the lateral orbitofrontal cortex, dorsolateral prefrontal cortex, cingulate cortex, and precuneus [63]. Although there is currently little research on the persistence of COIVD-19 outbreaks on college students’ mental health, some authors emphasize that the expected consequences of mental and physical health in the most vulnerable populations can be more or less predicted [64]. In view of the above situation, we should continue to pay attention to the mental health problems of college students in the post-epidemic period, and give continuous attention and psychological intervention treatment.

This study has several notable limitations: (1) The cross-sectional study could not analyze the causal relationship between sleep quality and depressive symptoms, and prospective cohort studies are needed to better understand the causal relationship in the future. (2) The measurement of sleep quality in this study used the PSQI questionnaire to conduct self-subjective evaluation, which is inevitably biased from the actual situation. In the future, more accurate wearable devices should be used for evaluation. (3) The sample of college students and the survey area are limited and need to be further expanded in the future.

## 5. Conclusions

In conclusion, our study identified an association between sleep quality and depressive symptoms among Chinese college students during the COVID-19 lockdown—that is, poorer sleep quality was associated with a higher incidence of depressive symptoms. Our research provides help for the government to formulate emergency public health policies to improve the sleep quality and depression symptoms of Chinese college students. Future research should be focused on interventions to prevent or reduce the development of depression symptoms. Educational campaigns such as obtaining good sleep habits are needed to improve the sleep quality of college students.

## Figures and Tables

**Table 1:** Characteristics of depressive symptoms in college students (%).

Variables	SDS							
	Males (*n* = 2862)				Females (*n* = 3833)			
	Depression	No Depression	Chi-Square	*p*	Depression	No Depression	Chi-Square	*p*
Sleep quality (PSQI)								
Good	31 (3.5)	856 (96.5)	840.956	<0.001	168 (16.5)	849 (83.5)	542.703	<0.001
Poor	1217 (61.6)	758 (38.4)			1664 (59.1)	1152 (40.9)		
Age (years)								
19	322 (39.0)	503 (61.0)	21.498	<0.001	697 (47.4)	775 (52.6)	4.595	0.204
20	381 (44.1)	483 (55.9)			575 (47.8)	627 (52.2)		
21	277 (42.3)	378 (57.7)			402 (50.4)	396 (49.6)		
22	268 (51.7)	250 (48.3)			158 (43.8)	203 (56.2)		
Urban and rural								
Urban	256 (38.9)	402 (61.1)	7.676	0.006	441 (46.3)	511 (53.7)	1.100	0.294
Rural	992 (45.0)	1212 (55.0)			1391 (48.3)	1490 (51.7)		
One-child family								
Yes	319 (34.2)	615 (65.8)	50.367	<0.001	275 (37.2)	464 (62.8)	41.095	<0.001
No	929 (48.2)	999 (51.8)			1557 (50.3)	1537 (49.7)		
BMI								
Underweight	126 (41.7)	176 (58.3)	2.275	0.517	380 (48.9)	397 (51.1)	3.826	0.281
Normal	672 (44.6)	834 (55.4)			982 (47.4)	1089 (52.6)		
Overweight	218 (41.3)	310 (58.7)			130 (43.3)	1270 (56.7)		
Obese	232 (44.1)	294 (55.9)			340 (49.6)	345 (50.4)		
Physical activity								
Yes	124 (32.0)	264 (68.0)	24.760	<0.001	94 (27.1)	253 (72.9)	65.561	<0.001
No	1124 (45.4)	1350 (54.6)			1738 (49.9)	1748 (50.1)		
Screen time								
≤2 h/d	249 (32.6)	514 (67.4)	50.926	<0.001	306 (35.3)	562 (64.7)	50.908	<0.001
>2 h/d	999 (47.6)	1100 (52.4)			1526 (51.5)	1439 (48.5)		
Sugar-sweetened beverages								
Yes	1024 (45.8)	1210 (54.2)	20.611	<0.001	1628 (49.2)	1678 (50.8)	20.215	<0.001
No	224 (35.7)	404 (64.3)			204 (38.7)	323 (61.3)		
Father’s education								
Elementary school and below	389 (49.7)	394 (50.3)	22.288	<0.001	506 (52.6)	456 (47.4)	24.402	<0.001
Middle school	767 (42.4)	1044 (57.6)			1186 (47.5)	1313 (52.5)		
College and above	92 (34.3)	176 (65.7)			140 (37.6)	232 (62.4)		
Mother’s education								
Elementary school and below	621 (46.2)	723 (53.8)	16.265	<0.001	861 (50.7)	837 (49.3)	59.420	<0.001
Middle school	575 (42.7)	772 (57.3)			932 (47.8)	1017 (52.2)		
College and above	52 (30.4)	119 (69.6)			39 (21.0)	147 (79.0)		
SES								
Low	277 (54.7)	229 (45.3)	37.815	<0.001	312 (58.8)	219 (41.2)	26.217	<0.001
Medium	834 (42.4)	1133 (57.6)			1299 (47.0)	1464 (53.0)		
High	137 (35.2)	252 (64.8)			221 (41.0)	318 (59.0)		
Total	1248 (43.6))	1614 (56.4)			1832 (47.8)	2001 (25.2)		

Note: SDS, self-rating depression scale; PSQI, Pittsburgh sleep quality index; BMI, body mass index; SES, socioeconomic status. *p*-values were obtained by the chi-square test. The division of different variables is described in the research methods section.

**Table 2:** The odds ratio of depressive symptoms for different variables by gender.

Variables	Depression Symptoms (SDS ≥ 50)							
	Males				Females			
	Adjusted OR	95%CI		*p*	Adjusted OR	95%CI		*p*
Sleep quality (PSQI)								
Good	1.00				1.00			
Poor	44.33	30.63	64.16	<0.001	7.30	6.09	8.76	<0.001
Age (years)								
19	1.00				1.00			
20	1.23	1.02	1.50	0.035	1.02	0.88	1.19	0.802
21	1.15	0.93	1.41	0.205	1.13	0.95	1.34	0.169
22	1.68	1.34	2.09	<0.001	0.87	0.69	1.09	0.222
Urban and rural								
Urban	1.00				1.00			
Rural	1.29	1.08	1.54	0.006	1.08	0.93	1.25	0.294
One-child family								
Yes	1.00				1.00			
No	1.79	1.53	2.11	<0.001	1.71	1.45	2.02	<0.001
BMI								
Underweight	1.00				1.00			
Normal	1.13	0.88	1.45	0.355	0.94	0.80	1.11	0.479
Overweight	0.98	0.74	1.31	0.903	0.80	0.61	1.05	0.101
Obese	1.10	0.83	1.47	0.505	1.03	0.84	1.26	0.781
Physical activity								
Yes	1.00				1.00			
No	1.77	1.41	2.23	<0.001	2.68	2.09	3.42	<0.001
Screen time								
≤2 h/d	1.00				1.00			
>2 h/d	1.88	1.58	2.23	<0.001	1.95	1.67	2.28	<0.001
Sugar-sweetened beverages								
No	1.00				1.00			
Yes	1.53	1.27	1.83	<0.001	1.54	1.27	1.85	<0.001
Father’s education								
College and above	1.00				1.00			
Elementary school and below	1.89	1.42	2.52	<0.001	1.84	1.44	2.35	<0.001
Middle school	1.41	1.07	1.84	0.013	1.50	1.20	1.87	<0.001
Mother’s education								
College and above	1.00				1.00			
Elementary school and below	1.97	1.40	2.77	<0.001	3.88	2.69	5.59	<0.001
Middle school	1.70	1.21	2.40	0.002	3.45	2.40	4.97	<0.001
SES								
High	1.00				1.00			
Low	2.23	1.70	2.92	<0.001	2.05	1.61	2.62	<0.001
Medium	1.35	1.08	1.70	0.009	1.28	1.06	1.54	0.011

Note: CI, confidence interval; OR, odds ratio; SDS, self-rating depression scale; PSQI, Pittsburgh sleep quality index; BMI, body mass index; SES, socioeconomic status. The division of different variables is described in the research methods section.

**Table 3:** The relationship between different factors of the PSQI and depressive symptoms by gender.

Variables	Depression Symptoms (SDS ≥ 50)							
	Males				Females			
	Adjusted OR	95%CI		*p*	Adjusted OR	95%CI		*p*
Subjective sleep quality								
Very good	1.00				1.00			
Fairly good	13.45	8.06	22.44	<0.001	6.11	4.43	8.44	<0.001
Fairly bad	113.20	66.13	193.77	<0.001	52.87	37.12	75.30	<0.001
Very bad	3516.28	1278.92	9667.67	<0.001	2557.61	913.87	7157.92	<0.001
Sleep latency								
≤15 min	1.00				1.00			
16–30 min	1.31	1.06	1.62	0.012	0.95	0.79	1.14	0.553
31–60 min	4.46	3.56	5.59	<0.001	3.74	3.09	4.51	<0.001
>60 min	23.66	17.35	32.26	<0.001	21.26	16.11	28.05	<0.001
Sleep duration								
>7 h	1.00				1.00			
6–7 h	1.56	1.18	2.07	0.002	1.70	1.37	2.11	<0.001
5–6 h	3.49	2.75	4.41	<0.001	2.35	1.94	2.86	<0.001
<5 h	6.80	5.39	8.58	<0.001	3.87	3.20	4.68	<0.001
Habitual sleep efficiency								
≥85%	1.00				1.00			
75–84%	2.80	2.18	3.60	<0.001	2.07	1.71	2.52	<0.001
65–74%	4.59	3.57	5.90	<0.001	3.57	2.92	4.37	<0.001
<65%	7.59	6.03	9.55	<0.001	4.40	3.66	5.30	<0.001
Sleep disturbance								
0	1.00				1.00			
1–9	2.65	2.10	3.33	<0.001	0.80	0.65	0.98	0.032
10–18	49.91	33.96	73.37	<0.001	21.05	15.88	27.89	<0.001
19–27	1026.30	324.50	3245.87	<0.001	805.04	255.98	2531.78	<0.001
Use of sleep medication								
Not during the past month	1.00				1.00			
Less than once a week	1238.22	593.09	2585.08	<0.001	733.50	397.43	1353.75	<0.001
Once or twice a week	2619.32	1101.02	6231.35	<0.001	1875.95	815.74	4314.11	<0.001
Three or more times a week	3213.16	1428.90	7225.40	<0.001	9601.10	2358.48	39084.97	<0.001
Daytime dysfunction								
No problem at all	1.00				1.00			
Only a very slight problem	3.81	2.80	5.18	<0.001	3.50	2.68	4.57	<0.001
Somewhat of a problem	6.19	4.56	8.39	<0.001	6.90	5.27	9.03	<0.001
A very big problem	40.16	28.51	56.57	<0.001	39.20	27.83	55.22	<0.001

Note: CI, confidence interval; OR, odds ratio; SDS, self-rating depression scale; PSQI, Pittsburgh sleep quality index. Sociodemographic factors, economics, and health-related factors were controlled for in the study analysis.

**Table 4:** Subgroup analysis of the relationship between sleep quality and depressive symptoms in college students with different variables.

Variables	Poor Sleep Quality									
	Males					Females				
	No Depression	Depression				No Depression	Depression			
	OR	OR	95%CI		*p*	OR	OR	95%CI		*p*
Age (years)										
19	1.00	39.03	19.67	77.46	<0.001	1.00	7.34	5.51	9.77	<0.001
20	1.00	43.98	22.89	84.51	<0.001	1.00	6.65	4.86	9.11	<0.001
21	1.00	78.33	28.60	214.56	<0.001	1.00	8.29	5.34	12.88	<0.001
22	1.00	33.02	15.66	69.63	<0.001	1.00	7.71	4.17	14.27	<0.001
Urban and rural										
Urban	1.00	30.02	15.01	60.05	<0.001	1.00	5.81	4.18	8.08	<0.001
Rural	1.00	50.32	32.50	77.93	<0.001	1.00	8.03	6.45	9.99	<0.001
One-child family										
Yes	1.00	58.85	25.84	134.03	<0.001	1.00	7.20	4.52	11.47	<0.001
No	1.00	40.86	26.95	61.97	<0.001	1.00	7.43	6.09	9.06	<0.001
BMI										
Underweight	1.00	49.62	17.51	140.58	<0.001	1.00	8.29	5.51	12.47	<0.001
Normal	1.00	39.32	24.43	63.28	<0.001	1.00	6.19	4.89	7.85	<0.001
Overweight	1.00	62.17	19.47	198.53	<0.001	1.00	16.67	6.48	42.88	<0.001
Obese	1.00	52.74	21.12	131.73	<0.001	1.00	8.71	5.57	13.62	<0.001
Physical activity										
Yes	——	——	——	——	——	1.00	2.33	1.42	3.82	0.001
No	1.00	35.36	24.36	51.33	<0.001	1.00	8.07	6.61	9.85	<0.001
Screen time										
≤2 h/d	1.00	40.15	21.36	75.46	<0.001	1.00	26.22	15.81	43.47	<0.001
>2 h/d	1.00	44.21	27.96	69.91	<0.001	1.00	4.89	4.00	5.97	<0.001
Sugar-sweetened beverages										
Yes	1.00	59.00	36.96	94.18	<0.001	1.00	8.16	6.63	10.05	<0.001
No	1.00	20.71	11.21	38.25	<0.001	1.00	4.25	2.85	6.33	<0.001
Father’s education										
Elementary school and below	1.00	27.06	15.32	47.79	<0.001	1.00	8.21	5.57	12.10	<0.001
Middle school	1.00	69.75	39.78	122.30	<0.001	1.00	7.10	5.70	8.84	<0.001
College and above	1.00	22.00	7.74	62.53	<0.001	1.00	6.20	3.42	11.25	<0.001
Mother’s education										
Elementary school and below	1.00	38.55	22.98	64.68	<0.001	1.00	7.76	5.83	10.34	<0.001
Middle school	1.00	53.79	30.48	94.91	<0.001	1.00	6.81	5.35	8.68	<0.001
College and above	1.00	28.13	6.54	120.92	<0.001	1.00	9.78	2.88	33.18	<0.001
SES										
Low	1.00	22.14	12.37	39.62	<0.001	1.00	12.49	6.84	22.81	<0.001
Medium	1.00	77.11	43.10	137.96	<0.001	1.00	7.52	6.05	9.35	<0.001
High	1.00	34.32	12.32	95.64	<0.001	1.00	4.17	2.77	6.28	<0.001

Note: CI, confidence interval; OR, odds ratio; BMI, body mass index; SES, socioeconomic status. ——, all the participants in the poor sleep quality group did not achieve the physical activity standard. The division of different variables is described in the research methods section. Sociodemographic factors, economics, and health-related factors were controlled for in the study analysis.

**Table 5:** The hierarchical logistic regression of depression symptoms for Chinese college students with different sleep quality.

Sleep Quality	Odds Ratio (95% Confidence Interval)		
	Crude Model	Model 1	Model 2
Males			
Good	1.00	1.00	1.00
Poor	44.33 (30.63, 64.16) ^a^	42.40 (29.23, 61.51) ^a^	43.81 (30.15, 63.65) ^a^
*p* for trend	<0.001	<0.001	<0.001
Females			
Good	1.00	1.00	1.00
Poor	7.30 (6.09, 8.75) ^a^	6.82 (5.66, 8.22) ^a^	6.78 (5.62, 8.18) ^a^
*p* for trend	<0.001	<0.001	<0.001
Total			
Good	1.00	1.00	1.00
Poor	12.92 (11.04,15.13) ^a^	12.04 (10.26,14.13) ^a^	12.01 (10.23,14.10) ^a^
*p* for trend	<0.001	<0.001	<0.001

Note: ^a^ indicate *p* < 0.01.

## Data Availability

Readers can obtain them from the corresponding author on reasonable request.
